# ﻿Resurrection of *Stipatremula* and taxonomy of the high-alpine species from the *Stipapurpurea* complex (Poaceae, Pooideae)

**DOI:** 10.3897/phytokeys.196.82598

**Published:** 2022-05-13

**Authors:** Marcin Nobis, Marta Krzempek, Arkadiusz Nowak, Polina D. Gudkova, Ewelina Klichowska

**Affiliations:** 1 Institute of Botany, Jagiellonian University, Gronostajowa 3, 30–387 Kraków, Poland Jagiellonian University Krakow Poland; 2 Institute of Biology, University of Opole, 45–052 Opole, Poland University of Opole Opole Poland; 3 Botanical Garden, Center for Biological Diversity Conservation, Polish Academy of Sciences, 02–976 Warszawa, Poland Center for Biological Diversity Conservation, Polish Academy of Sciences Warsawa Poland; 4 Research Laboratory ‘Herbarium’, National Research Tomsk State University, Lenin 36 Ave., 634050 Tomsk, Russia Tomsk State University Tomsk Russia; 5 Institute of Biology, Altai State University, Lenin 61 Ave., 656049, Barnaul, Russia Altai State University Barnaul Russia

**Keywords:** distribution, feathergrasses, hybridisation, *Lasiagrostis*, micromorphology, *Ptilagrostis*, taxonomy, typification

## Abstract

*Stipapurpurea* is a high-alpine species that occurs in cryophilous steppes, semi-deserts and stony slopes, from the Tian Shan and Pamirian Plateau through Qinghai-Xizang Plateau to the Himalayas and is characterised by a great morphological variability. During the revision of specimens of the taxon, we observed that the pattern of this variability is linked to the geographical distribution of the specimens. Numerical analyses (PCA and UPGMA) revealed three groups of OTUs corresponding to three morphotypes within the *S.purpurea* complex. A set of macro- and micromorphological characters, supported by a map of general distributional ranges, are presented to distinguish each of the three taxa within the complex and we reassess the status of *Lasiagrostistremula* described by Ruprecht in 1869. As a result, *Stipatremula*, *S.purpurea* and *S.arenosa* were distinguished within the complex. The intermediate characters of *S.arenosa* may suggest its putative hybrid origin (*S.tremula* × *S.purpurea*), whereas the presence of extremely long florets may be an expression of the gigas effect. We propose two new combinations (*S.tremula* and *S.arenosa*), describe a new nothospecies (*S.×ladakhensis*) that originated from hybridisation between *S.klimesii* and *S.purpurea* s.l. and designate the lectotype for *Ptilagrostissemenovii*. An identification key and detailed morphological description of species from the *S.purpurea* complex are also presented.

## ﻿Introduction

With ca. 11500 species divided among 750–770 genera (Kellogg 2015; Soreng et al. 2017; Hodkinson 2018), grasses (Poaceae) constitute one of the richest families of flowering plants, widely distributed and covering, as grasslands or bamboo forests, ca. 40% of the Earth’s land surface (Gibson 2009; Hodkinson 2018). Grasses occur on all continents and can be found in a wide variety of climates and habitats. Some species of grasses can grow in extreme habitats, such as hot or cold deserts and semi-deserts, grasslands and swards of polar areas or alpine meadows and steppes. Especially interesting are high-alpine species, which, due to strong resistance to cold, drought, wind, high ultraviolet radiation and nutrient poor soils, can grow in severe alpine environments and play an important role in the preservation and stabilisation of high mountain landscape diversity and heterogeneity. Examples of genera with species found in the highest areas of mountains may be: *Calamagrostis* Adans., *Colpodium* Trin., *Elymus* L., *Festuca* Tourn. ex L., *Helictotrichon* Besser, *Leymus* Hochst., *Ptilagrostis* Griseb., *Poa* L., *Puccinellia* Parl. and *Stipa* L. (Bor 1960, 1970; Tzvelev 1976; Cope 1982; Wu et al. 2006; Conti et al. 2020; Kellogg et al. 2020; Liu and Paszko 2020; Nobis et al. 2020).

Within the genus *Stipa* (feathergrasses), *S.purpurea* is an example of high-alpine species with a fairly wide distribution range, being dominant in alpine steppes and an important food component for herbivorous animals (Tzvelev 1968; Wu and Phillips 2006; Yue et al. 2011; Hu et al. 2015; Nobis et al. 2020). It was described by Grisebach (1868) from western Tibet. This species and several other alpine feathergrasses, such as *S.aliena* Keng, *S.basiplumosa* Munro ex Hook. f., *S.capillacea* Keng, *S.dickorei* M. Nobis, *S.klimesii* M. Nobis, *S.penicillata* Hand.-Mazz., *S.regeliana* Hack., *S.roborowskyi* Roshev., *S.subsessiliflora* (Rupr.) Roshev. and *S.zhadaensis* L.Q. Zhao & K. Guo (Nobis et al. 2014, 2016a, 2020; Zhao and Guo 2017), are distributed in the highest elevations of Central Asian mountains (Nobis et al. 2019a, 2020). *Stipapurpurea* occurs in alpine steppes and semi-deserts, stone slopes, gravel or sand terraces and valley sediments (Yue et al. 2011, Nobis et al. 2020) and is distributed from the Tian Shan and Pamir through Kunlun, Karakorum, Qinghai-Xizang Plateau to the Himalayas (Tzvelev 1968; Lu and Wu 1996; Wu and Wang 1999; Wu and Phillips 2006; Ma and Sun 2018; Nobis et al. 2020) at elevations between 1900 and 5240 m (Noltie 2000; Liu et al. 2008; Yue et al. 2011; Nobis et al. 2020). It is a dominant and diagnostic species of high alpine steppes and, in the north-eastern Qinghai-Tibetan Plateau, creates its own plant community, the so-called *Stipapurpurea* steppes (Yue et al. 2011). The species is also characterised by an extremely high morphological variability. In the past centuries, a several taxa that used to be included within the *S.purpurea* complex, namely, *S.purpurea*, *Lasiagrostistremula* Rupr., *Ptilagrostissemenovii* Krasn., *S.semenowii* Krasn., *S.semenovii* Krasn., *S.pilgeriana* K.S. Hao and S.purpureavar.arenosa Tzvel. were described (Grisebach 1868; Ruprecht 1869; Krasnow 1887a, 1887b, 1888; Hao 1938; Tzvelev 1968); however, almost all of them were later synonymised with *S.purpurea* (Roshevitz 1934; Tzvelev 1968, 1976; Kuo and Sun 1982, 1987; Freitag 1985; Wu and Phillips 2006; Nobis et al. 2020). The only exception is S.purpureavar.arenosa, which was accepted by some authors either in the rank of variety or subspecies within *S.purpurea* (Kuo and Sun 1982; Cui 1996; Lu and Wu 1996; Wu and Wang 1999; Nobis et al. 2016a). In recent years, some studies regarding morphological and genetic diversity of the species (Liu et al. 2008, 2016), seed variability (Li et al. 2015), adaptation to drought (Yang et al. 2015), grazing (Zhai et al. 2015) and prediction of distribution models (Ma and Sun 2018) were conducted. However, all of these were focused on populations of *S.purpurea* in the Qinghai-Tibet Plateau and there is a lack of studies that encompass the whole geographical range of the species. In all of the above-mentioned studies, the authors emphasised that *S.purpurea* is an extremely variable taxon, that varied morphologically in relation to latitude, longitude and altitude (Liu et al. 2008, 2016). The phenomenon of this variability within the geographical range is also well illustrated in the morphological descriptions of the species, which can be found in many identification keys, taxonomic elaborations or local floras. Besides the most variable characters, such as length of culms, length of leaf blades, the number of generative shoots or number of spikelets, which depend on local climate, elevation or grazing intensity in particular geographical regions, the variability is also noted within more conservative, species-specific characters, such as the length of ligules on the vegetative shoots, length of florets and length of glumes (cf. Ovchinnikov and Chukavina 1957; Tzvelev 1968; Bor 1970; Cope 1982; Freitag 1985; Kuo and Sun 1987; Cui 1996; Wu and Wang 1999; Noltie 2000; Wu and Phillips 2006).

During the preliminary revision of specimens representing *Stipapurpurea*, we confirmed the high morphological variability, especially regarding the plant height, the shape and length of ligules, the indumentum of lemma and the length of awns and glumes within specimens originating from different localities. We, furthermore, observed that the variability corresponds to the geographical distribution of particular morphotypes. Therefore, we performed a taxonomic revision of the *S.purpurea* complex across its entire geographical range to answer the questions: i) what is the morphological differentiation of the *S.purpurea* morphotypes within the geographical range of the taxon and which characters are the most conservative for them, ii) what are the differences in the distribution patterns of particular morphotypes within the complex, iii) what are the taxonomic relationships between the observed morphotypes and hitherto described taxa within the *Stipapurpurea* complex and iv) what is their taxonomic position?

## ﻿Methods

This study is based on plant material preserved in the following herbaria: AA, BM, CUH, E, GOET, K, KRA, KUN, LE, M, MSB, MW, MOIS, NY, P, PE, PR, TAD, TK, TASH (acronyms of the herbaria are used according to Index Herbariorum, Thiers 2022). Over 200 sheets with specimens belonging to *Stipapurpurea* complex (including all available types of taxa from the examined complex, i.e. *S.purpurea* – holotype and isotypes, *Lasiagrostistremula* – holotype, *Ptilagrostissemenovii* – lectotype and isolectotype, *S.pilgeriana* – holotype and S.purpureavar.arenosa – holotype and paratypes) were reviewed between 2009 and 2020. For comparison purposes, we also reviewed over 60 sheets with specimens representing *S.roborowskyi* and *S.klimesii*. The numerical analyses were based on 77 specimens from the *S.purpurea* complex (see specimens examined below). All specimens used in the analyses were mature and fully developed. Following the assumptions of numerical taxonomy, each specimen was considered as an operational taxonomic unit (OTU). Measurements were taken using a stereomicroscope (Nikon SMZ800) with a graduated scale eyepiece. Principal component analysis, based on the correlation matrix was used to characterise variation within and among taxa and extract the variables that best identify these taxa. Among 53 morphological characters (including: floret length, length of hairs on the ventral part of the lemma, length of hairs on the dorsal part of the lemma, lemma apex (glabrous/with corolla of hairs and length of hairs), callus length, callus base length and width, length of hairs on the dorsal part of the callus, length of hairs on the ventral part of the callus, awn length, lower segment of the awn length, middle segment of the awn length, terminal segment of the awn length, ratio terminal/lower + middle segment of the awn, width of the awn base, length of hairs on the lower segment of the awn, length of hairs on the middle segment of the awn, length of hairs on the terminal segment of the awn, ratio: length of hairs on the terminal segment of the awn/length of hairs on the lower segment of the awn, length of culms, number of culm nodes, distribution of nodes on the culm, length of ligule on the lower culm sheath, length of ligule on the middle culm sheath, length of ligule on the upper culm sheath, length of the lower glume, length of the upper glume, length of the longest ligules on the external leaf-sheaths on the vegetative shoot, length of the longest ligules on the internal leaf-sheaths on the vegetative shoot, character of the lower culm sheaths (glabrous/pubescent and length of hairs), character of leaf-sheaths on the vegetative shoots (glabrous/pubescent and length of hairs), length of panicle, width of panicle, length of the lower pedicles within the panicle, character of pedicles (flexuous/straight), no. of spikelets within the panicle, length of hairs on the adaxial surface of vegetative leaves, length of hairs on the adaxial surface of culm leaves, character of the abaxial surface of leaves (glabrous, scabrous, pilose), length of the vegetative leaves, length of the culm leaves) measured, scored or estimated, the most variable and important for the species identification and with high factor loadings revealed by the initial principal component analysis (PCA), were chosen for further analyses. In consequence, seven characters with factor loadings ≥ 0.65 (Table 1) were chosen for final PCA and ANOVA analyses. However, the results from all the biometric examinations are presented in morphological descriptions of the examined taxa. Subsequently, descriptive statistics of characters for all recognised groups were calculated. To reveal significant differences between means of characters across all examined groups (after using Levene’s test to assess the equality of variances), a one-way analysis of variance (ANOVA) and non-parametric Kruskal-Wallis test followed by post-hoc Tukey’s HSD test or multiple comparison test were calculated. The cluster analysis (based on the unweighted pair group method with arithmetic mean) was performed on the basis of seven characters (Table 1). The similarities among OTUs were calculated using Gower’s General Similarity Coefficient. The analyses were performed using Statistica 13 (StatSoft Inc. 2011) and PAST v. 3.12 (Hammer et al. 2001).

**Table 1.** Morphological characters used in the numerical analyses.

Abbreviation	Character	PCA	UPGMA
AL	Length of the floret (*anthecium*) (mm)	+	+
AwL	Length of the awn (mm)	+	+
CL	Length of the callus (mm)	+	+
C/S	Ratio: length of hairs on column to the length of hairs on seta		+
GL	Length of the lower glume (mm)	+	+
LCL	Length of ligules of the middle cauline leaves (mm)	+	+
LVL	Length of ligules of the vegetative shoots (mm)	+	+
SHL	Length of hairs on the lower cauline sheaths (mm)	+	

### ﻿Micromorphology

Micromorphological structures of the lemma, sampled from the middle parts of the panicles, were observed in examined species (three specimens per taxon). Samples were coated with gold using a JFC-1100E Ion sputter, manufactured by JEOL and photographed with a Hitachi S-4700 scanning electron microscope, at various magnifications.

## ﻿Results

### ﻿Numerical analysis

**Figure 1.**
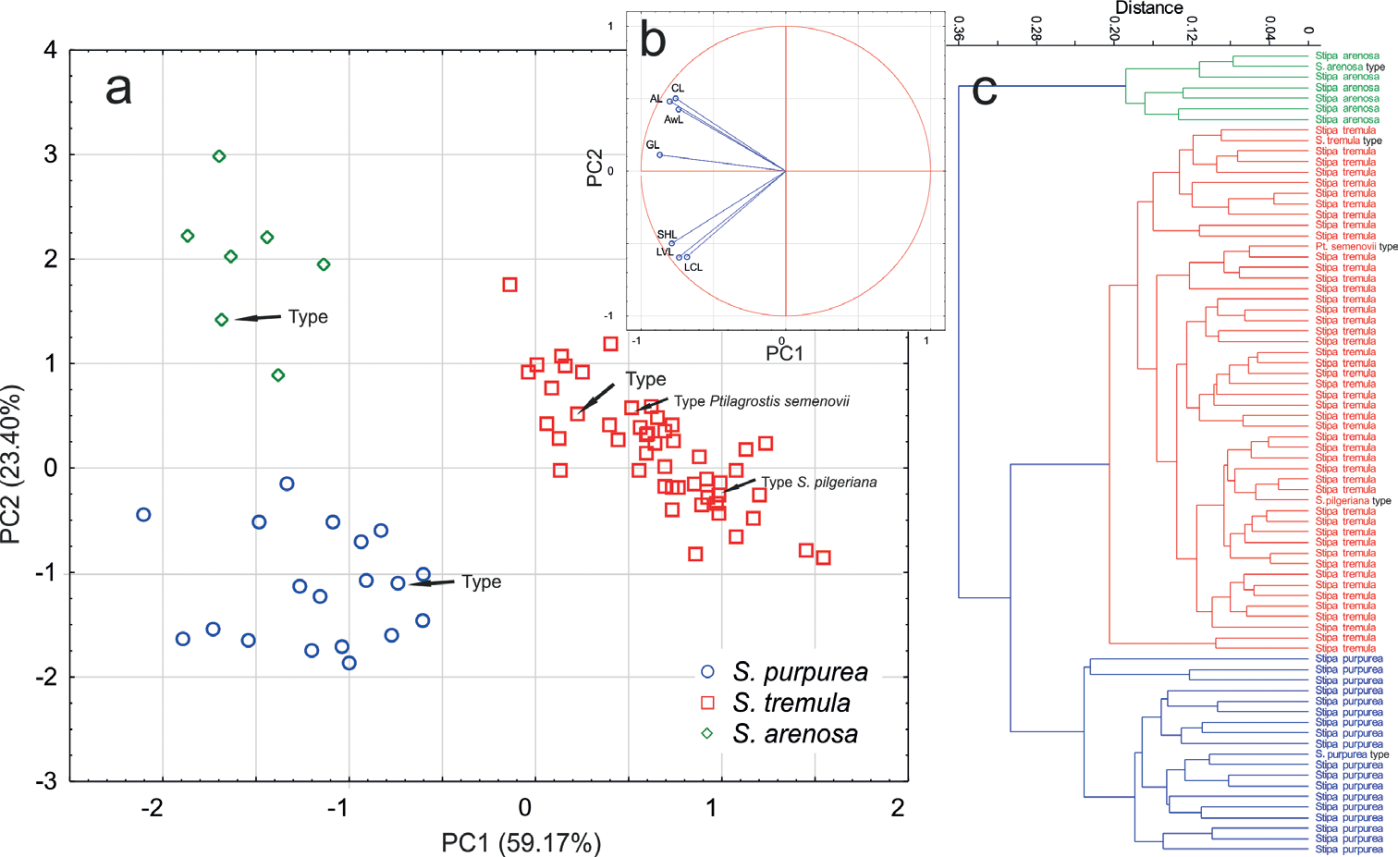
Biplot of the Principal Component Analysis performed on seven quantitative characters: (**a**) with projection of the variables on the factor plane PC1 × PC2; (**b**) and the cluster analysis UPGMA (**c**) of species from the *Stipapurpurea* complex.

The Principal Component Analysis (PCA) revealed that six of seven analysed characters have high factor loadings (r ≥ 0.7, Table 2). The first three components account for 88.23% of the total variation. The first component explains 59.17% of the variation, the second 23.4% and all of the analysed characters, i.e. AL, AwL, CL, C/S, GL, LCL, LVL, SHL, displayed high correlations with the first axis (Table 2). The scatter plot of the first two axes in PCA revealed three non-overlapping clusters of OTUs (Fig. 1), corresponding respectively to the typical specimens of *Stipapurpurea*, *S.tremula* and *S.arenosa* (see Taxonomic treatment below), whereas, the OTUs corresponding to types of *Ptilagrostissemenovii* and *Stipapilgeriana* were placed within the cloud of OTUs of *S.tremula*, what confirms their high morphological similarity to the letter taxon. The results of the one-way ANOVA/Kruskal-Wallis test revealed significant differences in all examined characters (Table 2) and the most significant values of F and H statistics obtained in the ANOVA and Kruskal-Wallis test were CL, LVL, GL and SHL. The results of the post-hoc tests (Tukey’s HSD test for variables with normal distribution and multiple comparison tests for characters with non-normal distribution) are presented in Table 2. Depending on the taxon, different characters were found to be significantly significant; however, all examined characters were suitable for distinguishing at least one pair of taxa. The greatest number of characters differentiated *S.purpurea* from *S.tremula*, whereas *S.arenosa* differentiated from *S.tremula* and *S.purpurea* by six and four characters, respectively (Table 2).

**Table 2.** Results of the Principal Component Analysis (PCA) of the *Stipapurpurea* complex, based on seven morphological characters (the highest factor loadings are in bold); one-way ANOVA with F and p values for characters with normal distribution and Kruskal-Wallis test with H and p values for characters with non-normal distribution (the highest F/H values are in bold); the post-hoc tests (Tukey’s HSD for characters with normal distribution and multiple comparison tests for characters with non-normal distribution): + – significant, p < 0.05, ns – not significant (abbreviations: *Stipapurpurea* – pur, *S.tremula* – tre, *S.arenosa* – are). For character abbreviations, see Table 1.

Character	PC1	PC2	PC3	F / H* value	*p* value	post-hoc test		
						pur-tre	pur-are	tre-are
AL	-**0.79**	0.49	0.18	35.65*	<0.05	+	ns	+
CL	-**0.75**	0.51	0.28	**50.10**	<0.05	+	+	+
AwL	-**0.73**	0.44	-0.44	23.81	<0.05	+	+	+
LCL	-0.69	-0.57	-0.18	29.92	<0.05	+	+	ns
LVL	-**0.74**	-0.57	0.06	**97.55**	<0.05	+	+	+
LG	-**0.86**	0.14	-0.11	44.97*	<0.05	+	ns	+
SHL	-**0.78**	-0.50	0.17	73.26*	<0.05	+	ns	+
Percent variation (%)	59.17	23.40	5.66					
No. of significant differences						7	4	6

**Figure 2.**
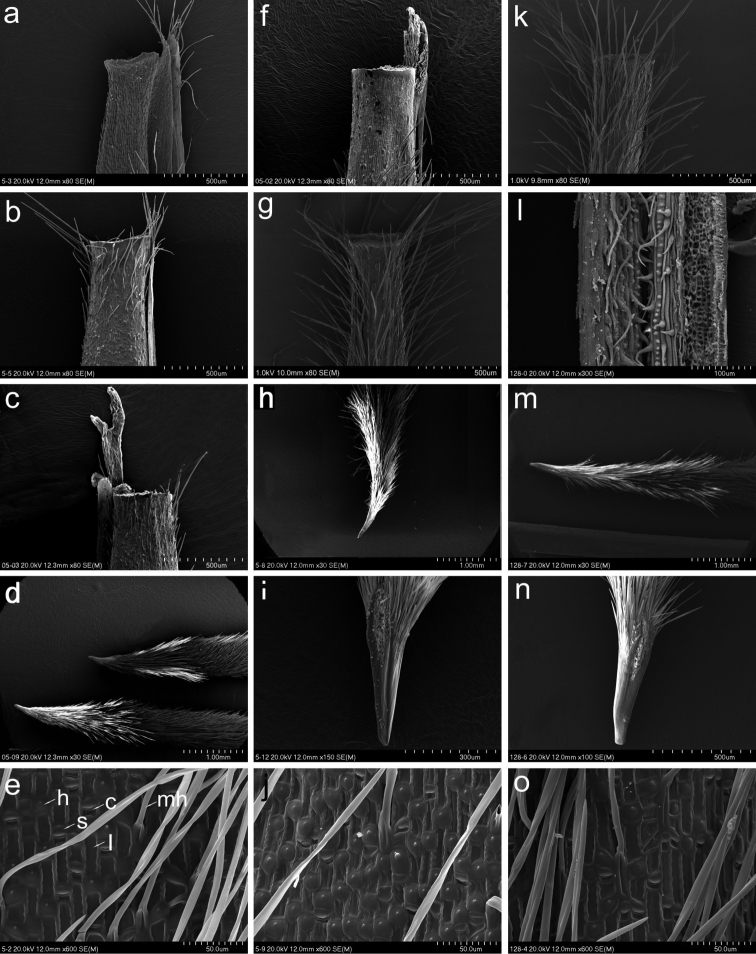
SEM morphology of *Stipatremula* (**a–e**), *S.purpurea* (**f–j**) and *S.arenosa* (**k–o**). Top of lemma (**a–c, f, g, k**), callus (**d, h, i, m, n**), adaxial surface of leaves (**l**), lemma epidermis (**e, j, o**). Abbreviations: c = cork cell, h = hook, l = long cell, s = silica body, mh = macro-hair. Vouchers: *S.tremula*; Kyrgyzstan, *Pimenov, Kmaishov, Kamsharaeva s.n.* (FRU), Tajikistan, *S.S. Ikonnikov 14896* (LE), China, Shinaihaixiang, *B. Paszko* (KRA); *S.purpurea*: India, *L. Klimeš 619* (KRA), China, *Maizang Team 76-7956* (PE); *S.arenosa*: China, *W.J. Roborowski 318* (KRA 476855), China, *B-z. Guo s.n*. (PE 707226).

Similarly to PCA, the cluster analysis (UPGMA) performed on the basis of seven characters (Table 2), also resulted in the delimitation of three clusters with OTUs belonging to *S.arenosa*, *S.purpurea* and *S.tremula* (Fig. 2). Within each of the revealed clades, the typical specimens for the three above-mentioned species are present (Fig. 2c). In this analysis, the OTUs of *Ptilagrostissemenovii* and *S.pilgeriana* were also located within the *S.tremula* clade.

### ﻿Micromorphology of the lemma epidermis

The representatives of the *Stipapurpurea* complex reveal a saw-like lemma epidermal pattern. In all three taxa, the fundamental long cells are rectangular to more or less square (Fig. 2e, j, o). The sidewalls of long cells are raised and undulate. Silica bodies are frequent and ovate to reniform and neighbouring with fairly frequent cork cells. Hooks are frequent and orientated towards the lemma apex, whereas prickles are rather sparse and, if present, occur mostly near the lemma apex. Macro-hairs are straight or bent near the base, (0.05–)0.15–0.80 mm long, cylindrical or string-like and twisted, with a bulbous base and a needle-like apex; they densely cover the lemma surface, from the bottom up to the top. However, the indumentum in the uppermost part of the lemma distinctly varies within and amongt examined species. In specimens of *S.purpurea*, the lemma can be covered by hairs up to the apex (Fig. 2g) or, at the distance of 0.2–0.5 mm to the lemma apex, it is glabrous, surpassed only (but not always) by minute 0.1–0.3 mm long apical lobes (Fig. 2f), whereas, in specimens of *S.tremula*, the lemma is either covered by hairs up to 0.5–1.7(–2.3) mm below the top and above being glabrous (Fig. 2a) or covered by hairs up to 0.5–1.7 mm below the top and above being glabrous, but at 0.2–0.5 mm below the top covered by scattered hairs 0.1–0.8 mm long, creating the corolla (Fig. 2b, c). In *S.arenosa*, all examined specimens have lemmas covered by hairs throughout, from the bottom to the top of the lemma (Fig. 2k).

### ﻿Distribution range

**Figure 3.**
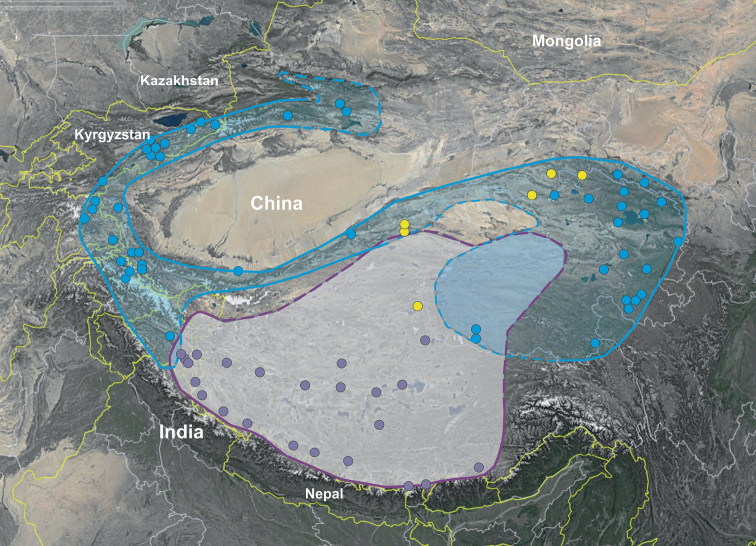
The distribution range of *Stipapurpurea* (purple backgrounds and points), *S.tremula* (blue backgrounds and points) and *S.arenosa* (yellow points).

The clouds of OTUs corresponding with the three examined species, namely *Stipapurpurea*, *S.tremula* and *S.arenosa*, are also well defined by the distribution patterns. The first two seem to be geographical vicariants occupying the highest elevations within the Central Asian Mountains. *Stipapurpurea* occurs mainly within alpine (cryophilous) steppes and semi-deserts, at altitudes between 4000 and 5200 m a.s.l. in south-western China (Xizang) and north India (Ladakh, Sikkim), whereas *S.tremula*, also a species of alpine steppes, occurs at somewhat lower altitudes, between (1900–)3000–4500 (–5100) m a.s.l. within the north-central Asian mountains, in Kyrgyzstan, Tajikistan, north Pakistan, India (Ladakh) and China (Xinjiang, Gansu, Qinghai, Sichuan, western and eastern Xizang; Fig. 3). The ranges of these taxa probably overlap on the area of south-western Qinghai and north-western Xizang and the range borders within the overlapping zones of both species need further studies. *Stipaarenosa* is the rarest taxon within the complex, known only from a few stands in central China (Fig. 3) in the contact zone between *S.purpurea* and *S.tremula*.

## ﻿Discussion

**Figure 4.**
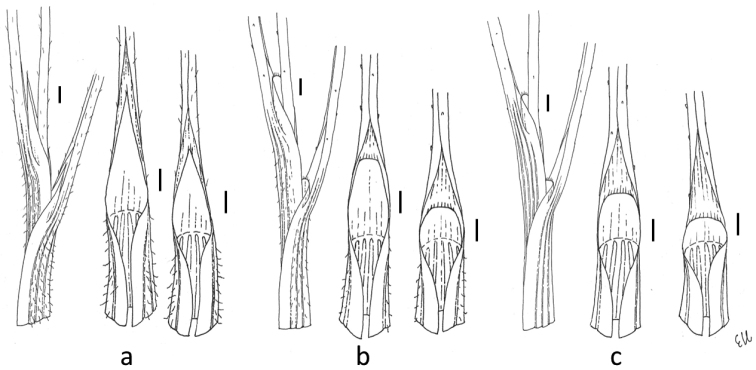
Morphology of the ligules of the internal and external sheaths/leaves of the vegetative shoots in **a***Stipapurpurea***b***S.arenosa***c***S.tremula*. Scale bars: 1 mm.

Although *Stipapurpurea* was described in the middle of the 19^th^ century and has been the subject of many different taxonomical, ecological, phytogeographical and molecular studies (Roshevitz 1934; Tzvelev 1968; Kuo and Sun 1987; Wu and Wang 1999; Wu and Phillips 2006; Liu et al. 2008, 2016; Li et al. 2015; Yang et al. 2015; Zhai et al. 2015; Ma and Sun 2018), to our knowledge, none of them referred to the whole geographical range of the species. Following the description of *S.purpurea* in different (regional) taxonomic treatments (Roshevitz 1934; Nikitina 1950; Ovchinnikov and Chukavina 1957; Kuo and Sun 1982, 1987; Wu and Phillips 2006), we can conclude that *S.purpurea* s.str. was misidentified with *S.tremula* and/or with *S.arenosa*. For instance, Kuo and Sun (1987), Cui (1996) and Wu and Wang (1999) identified the specimens with glumes 17–25 mm long and floret (= *anthecium*: callus+lemma) 12–14 mm long as S.purpureavar.arenosa, whereas the plants with glumes up to 13–17 mm long, floret 8–10 mm long (and glabrous leaf sheaths, Wu and Wang 1999) as S.purpureavar.purpurea. Information on the length of ligules on the vegetative shoots are usually not present in morphological descriptions (in Wu and Phillips 2006, the ligules are described as being ca. 1 mm long). The ligules presented on the schematic figures are rather short (ca. 2 mm long in Kuo and Sun 1982, 1987 and Cui 1996) and triangular (somewhat similar to those in *S.tremula*) instead of long and acute as, in fact, are present in *S.purpurea* s.str. (Fig. 4).

*Stipapurpurea* was transferred by Roshevitz (1934) to the genus *Ptilagrostis* as *P.purpurea* (Griseb.) Roshev.; however, this affiliation was later rejected by the other taxonomists (e.g. Pazij 1968; Tzvelev 1968, 1976; Bor 1970; Kuo and Sun 1987). The representatives of the examined species from the *S.purpurea* complex reveal a saw-like lemma epidermal pattern, that is typical for representatives of the genus *Stipa* rather than those belonging to *Ptilagrostis* (Barkworth and Everett 1987; Romaschenko et al. 2012; Nobis 2013, 2014; Nobis et al. 2016b, 2019a, 2019b, 2020), whereas the presence of fairly frequent cork cells on the lemma epidermis makes the species more similar to the high mountain, so-called Himalayan feathergrasses from sections *Regelia* Tzvel. or *Pseudoptilagrostis* Tzvel., i.e. *S.regelii*, *S.aliena*, *S.dickorei*, *S.subsessiliflora*, *S.penicillata*, *S.klimesii* or *S.roborowskyi* rather than those from section Barbatae A. Junge (Nobis et al. 2015, 2016a, 2019b, 2020), to which the species used to be included (Tzvelev 1974, 1976). This above-mentioned close relationship was also confirmed in molecular phylogenetic studies (Hamasha et al. 2012; Romaschenko et al. 2012; Krawczyk et al. 2017; Nobis et al. 2019c), where *S.purpurea* is placed in one common clade together with other Himalayan species of feathergrasses.

### ﻿Resurrection of *Stipatremula*

*Stipatremula* was described by Ruprecht (1869) as *Lasiagrostistremula* and synonymised with *S.purpurea* or with *Ptilagrostispurpurea* by subsequent agrostologists (Roshevitz 1916, 1934; Pazij 1968; Tzvelev 1968, 1976; Bor 1970; Freitag 1985; Kuo and Sun 1987; Wu and Phillips 2006; Nobis et al. 2017, 2020). However, our morphological analysis revealed a set of characters, including the shape and length of ligules of both the vegetative shoots and cauline leaves, characters of sheaths of the vegetative shoots and the lower cauline leaves as well as length of glumes (Fig. 4, Table 3), that led us to distinguish it from *S.purpurea* s.str. and reassess the status of this taxon.

**Table 3.** A comparison and differences in the main morphological characters of the species from the *Stipapurpurea* complex and allies.

Taxon Character	*Stipazhadaensis*	*Stiparoborowskyi*	*Stipaklimesii*	*Stipa×ladakhensis*	*Stipatremula*	*Stipaarenosa*	*Stipapurpurea*
Character of sheaths of vegetative shoots	densely pubescent	glabrous and smooth	densely pubescent	densely pubescent	glabrous and smooth	densely pubescent	densely pubescent
The longest ligules on the vegetative shoots [mm]	2–3	0.5–1.5(–2)	(2–)3.5–8(–10)	(1.5–)2–4(–5.5)	(0.5–)1–2(–3)	(1–)1.5–3(–4.5)	(3.8–)4.5–6.5(–8)
Shape of ligules on the vegetative shoots	truncate	truncate	acute	acute	truncate	truncate	acute
Ligules of middle cauline leaves [mm]	ca. 2	(1–)1.5–2.5(–3.5)	(1.5–)2.5–8(–9)	2–4(–5)	(1–)1.5–2.7(–4)	(1.6–)1.9–3.5(–4.5)	(2.2–)4.5–8(–9)
Character of lower cauline leave sheaths	pubescent	glabrous and smooth	densely pubescent	densely pubescent	glabrous and smooth	sparsely pubescent to glabrous	densely pubescent
Panicle	+/- lax	compressed	compressed	+/-compressed	lax	lax	lax
Branches of the panicle	straight	straight	straight	upper straight, lower slightly flexuous	flexuous	flexuous	flexuous
Glumes [mm]	20–28	11–15	13–18	16–19(–20)	(13–)14–17(–19.5)	(19.5–)21–23.5(–25)	(17–)19–26(–28)
Floret [mm]	8–11	(6–)6.5–7.5(–8)	(7–)8.3–9.5(–10.5)	8.5–10.2	(7.5–)8.4–9.5(–11)	(11.5–)12–14(–15)	(8.5–)9.5–11.2(–12.1)
Callus [mm]	ca. 1.5	1.2–2	1.4–2	1.5–1.9	(1.5–)1.6–2(–2.3)	(2–)2.3–3(–3.8)	(1.8–)2–2.4(–2.7)
Awn [mm]	60–90	(40–)47–60(–68)	(25–)35–45(–52)	46–58	(44–)60–74(–90)	(78–)95–112(–130)	(58–)70–91(–105)
Hairs on lower segment of awn (column) [mm]	(0.6–)1–1.5	1.5–2.1	(1.3–)1.5–2(–2.4)	1.7–2.3	(1.3–)1.7–2(–2.3)	(1.5–)1.6–2(–2.3)	(1.5–)1.6–2(–2.2)
Hairs on seta [mm]	0.5–1	(0.3–)0.5–1.1(–1.4)	(1–)1.3–2(–2.3)	1.6–2.1	(1.8–)2.2–2.6(–3.1)	(2.1–)2.3–2.5(–3)	(1.9–)2.1–2.4(–2.7)
Length of hairs on seta to the length of hairs on column	shorter	shorter	shorter	shorter to equal	longer	longer	longer

Within the *S.purpurea* complex, the two additional species, *Ptilagrostissemenovii* (≡ *S.semenovii* and *S.semenowii*) and *S.pilgeriana*, were described respectively by Krasnow [spelled also as Krassnoff or Krassnow] (1887a, 1887b, 1888 [three times!]) from Kyrgyzstan and by Hao (1938) from Gansu in China. However, morphologically, neither taxon differs from *S.tremula* in any of the examined characters. In our analyses, the OTUs of both type species are located in the central part of the cloud within the OTUs of *S.tremula* (Fig. 1); thus, we treat both of them as conspecific with the last-mentioned species.

### ﻿On the origin of *Stipaarenosa*

*Stipaarenosa* was described in the rank of variety as S.purpureavar.arenosa by Tzvelev (1968), who distinguished it from the typical variety, based on the longer glumes (17–22 vs. 12–17 mm long), longer florets (12–13 vs. 8–11 mm long) and longer awns (8–9 vs. 6–8.5 mm long). In our morphological analyses, the OTUs of *S.arenosa* were clustered separately from those of *S.purpurea* and *S.tremula*. The main characters that distinguish *S.arenosa* from *S.purpurea* and *S.tremula* are the length of the floret and the length of callus (Table 3). The other characters are rather intermediate between *S.purpurea* and *S.tremula*. Unlike *S.tremula*, *S.arenosa* has much longer glumes, 19.5–25 mm long and more or less pubescent sheaths of both vegetative and cauline leaves, which makes it similar to *S.purpurea*; however, the presence of fairly short and truncate ligules makes it more similar to *S.tremula* than to *S.purpurea* (Tables 2, 3). These characters may suggest that the taxon originated from hybridisation between *S.tremula* and *S.purpurea*, whereas the presence of such long florets (longer than in both putative parental species) may be a result of genome duplication, which sometimes happens in hybrids and is called as the Gigas effect (Stebbins 1971; Levin and Donald 2002; Soltis et al. 2014; Meeus et al. 2020). Nevertheless, the putative hybrid origin of *S.arenosa* requires confirmation using karyological and integrative morpho-molecular methods, such as those used recently to confirm the origin of other hybrids and cryptic taxa of feathergrasses (Nobis et al. 2019c; Baiakhmetov et al. 2020, 2021; Nie et al. 2020; Tkach et al. 2021).

## ﻿Taxonomic treatment

### ﻿A key to the identification of species from the *Stipapurpurea* complex

**key 1**

**Table:** 

1	Sheaths of the vegetative shoots and the lower cauline leaves always glabrous and smooth, glumes (13–)14–17(–19.5) mm long	***S.tremula***
–	Sheaths of the vegetative shoots and the lower cauline leaves more or less densely and shortly pubescent (rarely almost glabrous), glumes (17–)19–26(–28) mm long	**2**
2	Floret (*anthecium*) (8.5–)9.5–11.2(–12.1) mm long, the longest ligules on the vegetative shoots (3.8–)4.5–6.5(–8.0) mm long, acute with a few cilia at the apex, sheaths of the lower cauline leaves always densely and shortly pubescent	***S.purpurea***
–	Floret (11.5–)12–14(–15) mm long, the longest ligules on the vegetative shoots (1.0–)1.5–3.0(–4.5) mm long, truncate to slightly acute and ciliate on the apex, sheaths of the lower cauline leaves more or less densely and shortly pubescent rarely almost glabrous	***S.arenosa***

**key 2**

**Table:** 

1	Sheaths of the lower cauline leaves densely and shortly pubescent, the longest ligules on the vegetative shoots (3.8–)4.5–6.5(–8.0) mm long, acute with a few cilia at the apex	***S.purpurea***
–	Sheaths of the lower cauline leaves glabrous and smooth or rarely sparsely and shortly pubescent, the longest ligules on the vegetative shoots (0.5–)1.0–3.0(–4.5) mm long, truncate to slightly acute and ciliate at the apex	**2**
2	Floret 7.5–11 mm long, sheaths of the vegetative shoots and the lower cauline leaves always glabrous and smooth, glumes (13–)14–17(–19.5) mm long, the longest ligules on the vegetative shoots (0.5–)1.0–2.0(–3.0) mm long, truncate and ciliate on the apex	***S.tremula***
–	Floret (11.5–)12–14(–15) mm long, sheaths of the vegetative shoots and the lower cauline leaves more or less densely and shortly pubescent (rarely almost glabrous), glumes 19.5–25 mm long, the longest ligules on the vegetative shoots (1.0–)1.5–3.0(–4.5) mm long, truncate to slightly acute and ciliate on the apex	***S.arenosa***

#### ﻿ Stipa purpurea

Taxon classificationPlantaePoalesPoaceae

Griseb., Nachrichten von der Königlichen Gesellschaft der Wissenschaften und der Georg-Augusts-Universität zu Göttingen 3: 82–83. 1868.

22143B86-EC9B-5704-A6E2-A559A4FA2BB9

≡Ptilagrostispurpurea (Griseb.) Roshev., Fl. URSS 2: 76. 1934

##### Type protologue.

T. Nari Khorsum, *H. v. Schlagintweit*. **Type**: [China] Tibet, Gnari (Nari) Khorsum, alt. 5000 m, 5–15 Sep 1855, *Schlagintweit 7116* (holotype, GOET!; isotypes, BM 959325!, K 000032088!, LE 9281!, P!).

##### Description.

***Perennial plants***, densely tufted, with a few culms and numerous vegetative shoots; culms (11.5–)16.0–37.2(–60.0) cm tall, 1–2(–3)-noded, nodes distributed close together in the lowermost part of the culm. ***Leaves of vegetative shoots***: sheaths shortly and densely pubescent; ***ligules*** acute, with few cilia at the apex, on the external sheaths (1.5–)3.5–4.5(–6.0) mm long, whereas on the internal sheaths (3.8–)4.5–6.5(–8.0); ***blades*** convolute, green, pale green to greyish, (4.2–)6.0–14.0(–20.0) cm long, 0.3–0.5(–0.7) mm in diameter, adaxial surface covered by dense, 0.15–0.25 mm long hairs, abaxial surface glabrous, scabrous or shortly pilose (grading to almost smooth towards the apex). ***Cauline leaves***: lower sheaths densely and shortly pubescent, middle and upper sheath shortly pubescent or glabrous; ***ligules*** acute, on the lower sheaths (2.0–)4.0–7.2(–8.0) mm long, on the middle sheaths (2.2–)4.5–8.0(–9.0) and on the upper sheaths (3.5–)4.8–8.1(–9.2); ***blades*** convolute, green, pale green or greyish (4.5–)4.7–6.1(–7.8) cm long, adaxial surface covered with short hairs, abaxial surface glabrous, scabrous to shortly pubescent. ***Panicle*** (3.5–)9.7–17.5(–20.0) cm long, open, with (3–)6–15(–20) spikelets, at base enclosed by the sheath of the uppermost leaf; branches ascending, flexuous setulose, slightly scabrous, to glabrous and smooth, single or paired, 2–6 cm long. ***Glumes*** subequal (the lower slightly longer than the upper), purplish, (17–)19–26(–28) mm long, lanceolate, with hyaline margins and long tip. ***Floret*** (lemma + callus) (8.5–)9.5–11.2(–12.1) mm long and up to 1.0 mm wide. ***Callus*** (1.8–)2.0–2.4(–2.7) mm long, densely pilose, on ventral part with hairs (0.4–)0.6–0.8(–1.0) mm long, on dorsal with (0.4–)0.5–0.7(–0.8) mm long hairs; callus base 0.5–0.8 mm long and 0.15–0.20 mm wide, sharply pointed, scar narrow-elliptic. ***Lemma*** coriaceous, straw-coloured, purplish or brownish, covered throughout (from the bottom to top) by dense ascending to appressed hairs 0.3–0.8 mm long or the uppermost part of lemma, at 0.2–0.5 mm to the top, completely glabrous (hairless); ***top of lemma*** glabrous or surpassed by a ring of unequal hairs 0.2–0.8 mm long and with (or without) two minute apical lobes 0.1–0.3 mm long. ***Awn*** (58–)70–91(–105) mm long, bigeniculate; ***the lower segment of the awn*** (column) (6.5–)10–13(–16) mm long, twisted, 0.3–0.4 mm wide near the base, with (1.5–)1.6–2.0(–2.2) mm long hairs, ***the middle segment*** of the awn (7–)8–9(–10) mm long, twisted, with (1.7–)2.0–2.3(–2.5) mm long hairs; ***terminal segment*** (seta) slightly arcuate or flexuous (40–)50–69(–80) mm long with hairs longer than those on the column, (1.9–)2.1–2.4(–2.7) mm long, gradually decreasing in length towards the apex. ***Palea*** equalling lemma in length. ***Ovary*** with two styles.

##### Habitat.

High mountain steppes, semi-deserts, stony slopes, gravelly or sandy flats and valley silt, 4000–5200 m a.s.l.

##### Distribution.

Himalayas, southern Karakorum, Tibetan Plateau (Fig. 3); China (Xizang), India (Ladakh, north Sikkim) (Noltie 2000; Liu et al. 2008; Ma and Sun 2018).

##### Selected specimens examined.

**China: Xizang Province**, Rebang, Ritu County, alt. 4300 m, 28 Aug 1976, *Xizang Team 76-9126* (PE); east of Ando County, around transit station 23, flat landscape, 3 Aug 1961, *S. Wang 3698* (PE); Ali District, Gaer County, Menshi, gritty hillside, 9 Jul 1976, *Maizang Team 76-7956* (PE); Aligaize County, plateau, alt. 4450 m, Aug 1978, *F. Li 015* (PE 707342); Geji County, hillside, *no. 13545* (PE); Zuozuo District, Gar County, Langjiu, Ali, alt. 4650–4700 m, grassland, 10 Aug 1976, *Qing Zang team 76-8646* (PE 707397); alt. 4600 m, 17 Sept 1976, *no. 10333* (PE 707387); alt. 4700–5000 m, 1 Sept 1976, *no. 10106* (PE 707334); alt. 5000 m, 19 Jul 1976, *Xizang team 76-8541* (PE 707339); alt. 4900 m, 21 Aug 1976, *Xizang team 76-9102* (PE 707338); alt. 4400 m, 9 Jul 1976, *Xizang team 76-7956* (PE 707336); alt. 4800 m, 24 Jul 1976, no. 9819 (PE 707332); alt. 5100 m, 13 Sept 1976, *no. 9032* (PE 707337); Xizang Province, alt. 5050 m, 1 Aug 1976, no. 4900 (PE 707390); alt. 5000 m, 17 Aug 1976, no. 10025 (PE 707393); Ritu County, Rebang, gritty land, alt. 4300 m, 28 Aug 1976, *Xizang team 76-9126* (PE 707345); Purang County, Huoer, north slope, hillside grassland, alt. 4860 m, 22 Jul 1976, *Qing zhang team 76-8569* (PE). **India**: NW India, Jammu and Kashmir State, Ladakh, Rupshu, Samad Rokchen, Valley to Rang, alt. 4810–4900 m, 33°15.2'N, 78°05.7'E, 5 Aug 2001, *L. Klimeš 1262,1263* (KRA); NW India, Jammu and Kashmir State, Ladakh, Rupshu, Tso Moriri, slopes along the Luglung River, alt. 5200 m, 23 Aug 1999, 33°2'N, 78°27'E, *L. Klimeš 619* (KRA); NW India, Jammu and Kashmir State, Ladakh, Rupshu, Samad Rokchen, crossing Thukje - Polokongka, Polokongka - Nuruchan, alt.4630–4660 m, 5 Aug 2001, 33°16.6'N, 78°4.6' E, *L. Klimeš 1260* (KRA 479102); Sikkim, Naku La, alt. 16000 ft, 2 Nov 1909, *Ribu & Rhomoo 2769* (CUH).

#### ﻿ Stipa tremula

Taxon classificationPlantaePoalesPoaceae

(Rupr.) M. Nobis comb. nov.

6A07731E-EC33-5D11-BD74-D81320BA5C88

urn:lsid:ipni.org:names:77297804-1

= Ptilagrostissemenovii Krasn. [originally P.semenovi Krassn.], Spisok rastenii sobrannykh v vostochnom Tyan-Shane, letom 1886 goda, 125, 1887. Type protologue: Prope fl. sary-Jassy. Type: Ptilagrostistianschanica Krassn., Ad flumen Sary-Jassy, 1 Aug 1886, *Krassnow s.n.* (lectotype, distinguished here, LE 01009431!, isolectotype, LE 01009430!);
≡ Stipasemenowii Krasn. [originally S.semenowi Krassn.], Scripta Botanica Horti Universitatis Imperialis Petropolitanae, Botanicheskiia Zapiski 2(1): 22. 1887. Type protologue: In valle fluminis Sary-Jassy in montibus Thian-Schan non procul ab alpe Chan-tengri et in trajectu Turguen-Aksu non rara;
≡ Stipasemenovii Krasn. [originally S.semenovi Krassn.], Zapiski Imperatorkago Russkago Geograficheskago Obschestva, Opyt’ istorii rasvitya flory yuzhnoi chasti vostochnago Tyan-Shanya 19: 341–342. 1888. Type protologue: In valle fluminis Sary-Jassy in montibus Thian-Schan non procul ab alpe Chan-tengri et in trajectu Turguen-Aksu non rara.
= Stipapilgeriana K.S. Hao, Botanische Jahrbücher für Systematik, Pflanzengeschichte und Pflanzengeographie 68(5): 583–584. 1938. Type protologue: China: Kokonor [Qinghai]: Ming-ke-Shan, Tsi-gi-gen-ba-Gebiete, 3900 m (Nr. 1009 – am 25 August). Type: Kokonor [Qinghai], Mingke, Tsigigenpa, alt. 3900 m, 25 Aug 1930, *Hopkinson 1009* (holotype, PE 707247 [additional no. on the sheet 01940135], [label 2: S.pilgeriana Hao sp. nov.]).

##### Basionym.

Lasiagrostis (Leptanthele) tremula Rupr., in Ost.-Sack. & Rupr., Sertum Tianschanicum, Mémories de L’Académie Impériale des Sciences de St.-Pétersbourg, Sér. 7, 14(4): 35. 1869.

##### Type protologue.

Die Gegend des Sarymeki-Flüsschens, 28 Jul 1867, *F. Osten-Sacken s.n*. **Type.** [China] In der Gegend des Flusses Sarymeki, Südlicher Abhang des Tian-Schan, in regione subalpina jugi Thian-Schan, 28/9 Julio 1867, Lib. Baro *Fr. Osten-Sacken s.n*. (lectotype, designated by Tzvelev 1976: 583, LE 01009426!, isolectotypes, P 02241060!, K 000587435!).

##### Description.

***Perennial plants***, densely tufted, with a few culms and numerous vegetative shoots; culms (11–)20–30(–45) cm tall, 1–2(–3)-noded, nodes distributed close together and only in the lowermost part of the culm. ***Leaves of vegetative shoots***: sheaths glabrous and smooth rarely sparsely and shortly pubescent; ***ligules*** truncate, ciliate on the apex, on the external sheaths (0.3–)0.7–1.1(–1.6) mm long, whereas on the internal sheaths, (0.5–)1.0–2.0(–3.0) mm long; ***blades*** convolute, green, pale green to greyish, (3–)4–12(–18) cm long, 0.3–0.5(–0.7) mm in diameter, adaxial surface densely covered by 0.15–0.25 mm long hairs, abaxial surface glabrous or scabrous to shortly pilose and grading to almost smooth towards the apex. ***Cauline leaves***: sheaths glabrous and smooth; ***ligules*** on the lower sheaths truncate or acute (0.4–)1.0–1.5(–2.1) mm long, on the middle and upper sheaths acute (1.0–)1.5–2.7(–4.0) and 2.0–3.1(–3.6), respectively; ***blades*** convolute, green, pale green or greyish (2.3–)2.0–3.0(–8.9) cm long, adaxial surface covered with short hairs, abaxial surface glabrous, scabrous to shortly pubescent. ***Panicle*** (7–)11–18(–26) cm long, open, with (3–)6–15(–18) spikelets, at base enclosed by the sheath of the uppermost leaf or rarely exerted; ***branches*** ascending, flexuous, setulose, slightly scabrous to glabrous and smooth, single or paired, 2–6 cm long. ***Glumes*** subequal (the lower slightly longer than the upper), purplish, (13.0–)14–17.0(–19.5) mm long, lanceolate, with hyaline margins and long tip. ***Floret*** (lemma + callus) (7.5–)8.4–9.5(–11.0) mm long and (0.7–)0.8–1.0 mm wide. ***Callus*** (1.5–)1.6–2.0(–2.3) mm long, densely pilose, on ventral part with hairs (0.4–)0.5–0.8(–1.0) mm long, on dorsal with (0.3–)0.4–0.6(–0.8) mm long hairs; callus base 0.5–0.8 mm long and 0.15–0.25 mm in diameter, sharply pointed, scar narrowly elliptic. ***Lemma*** coriaceous, pale-green, purplish or brownish, covered throughout, from the bottom up to 0.5–2.3 mm to the top, by dense ascending to appressed hairs 0.3–0.8 mm long, above being glabrous (hairless) or with scattered hairs just below the apex; ***top of lemma*** glabrous or surpassed by a poorly- to well-developed ring of unequal hairs 0.2–0.5 mm long and with (or without) two minute apical lobes. ***Awn*** (44–)60–74(–90) mm long, bigeniculate; ***the lower segment of the awn*** (column) (6–)11–15(–20) mm long, twisted, 0.3–0.4 mm wide at the base, with (1.3–)1.7–2.0(–2.3) mm long hairs, ***the middle segment of the awn*** (5–)6.5–10(–13) mm long, twisted, with (1.6–)2.0–2.3(–2.6) mm long hairs; ***terminal segment of the awn*** (seta) slightly arcuate or flexuous (26–)39–53(–73) mm long with hairs longer than those on columns, (1.8–)2.2–2.6(–3.1) mm long, gradually decreasing in length towards the apex. ***Palea*** equalling lemma in length. ***Ovary*** with two styles.

##### Habitat.

High mountain steppes, dry grasslands, mats, screes, semi-deserts, stony slopes, gravelly or sandy flats and valleys (1900–)3000–4800(–5100) m a.s.l.

##### Distribution.

Tian Shan, Pamir, Karakorum, eastern Himalayas, Kun-lun, Qilan-Shan, eastern Tibetan Plateau (Fig. 3); Kazakhstan, Kyrgyzstan, Tajikistan, north Pakistan, India (Ladakh), China (Xinjiang, Gansu, Qinghai, Sichuan, western and eastern Xizang) (Pavlov 1956; Ovchinnikov and Chukavina 1957; Lazkov and Sultanova 2014).

##### Note.

*Lasiagrostistremula* was described from Tian-Shan Mts. by Ruprecht (in Osten-Sacken and Ruprecht 1869), based on the collection of Fr. Osten-Sacken (without indication of type, place of its preservation and the collection number). During the revision of the herbarium material of *Stipapurpurea* s.l., we found three sheets with specimens of *Lasiagrostistremula* collected by Osten-Sacken, of which the one preserved in LE, previously regarded as type by Tzvelev 1976, Freitag 1985 and Nobis et al. 2020) is here corrected to lectotype. The duplicates preserved in K and P are isolectotypes.

##### Selected specimens examined.

**Kyrgyzstan**: Tsentralnyi Tyan’-Shan’, dolina r. Kuilyu bliz ust’ya r. Oroi-su, na rechnoi terrase, alt. 2100 m, 10 Aug 1956, *I.S. Pushkin s.n.* (MW 803233, 803232); Tian-Schan centr., in valle flum. Sary-tschat, prope glaciem Kolpakovskyi, orientem versus a statione meteorologica, steppum frigidum, alt. ca. 3400 m, *D. Wyschiwkin s.n.* (MW 803231, US 04002929, P 02662772); Narynskii raion, bassein r. Aksai, khr. Kok-kiya, kobrezovyi lug, 15 Aug 1926, *M. Sovetkina, M. Uspenskaya 1745* (MW 803 229); Issykulskie syrty, kovyl’. step, alt. ca. 4000 m, 16 Aug 1953, *N. Trulevich s.n*. (MW 803234); Issykulskie syrty, kovyl’. step, alt. ca. 3000 m, 16 Aug 1953, *N. Trulevich s.n*. (MW 803228); Semirech. obl. Przhevalsk. u. dolina r. Naryna mezhdu r. Bashka-Su i r. Ulan’, kamenistye sklony, 20 Jul 1913, *V. Sapozhnikov 252* (LE); Semirech. obl. Przhevalsk. u. r. Ak-tash’, plato, alpiiskaya pustyn. step’, 28 Jul 1913, *V. Sapozhnikov 250* (LE, TK); Issyk-Kulskaya obl., Ak-Sulskii raion, khr. Teskei Ala-Too, pravyi bereg r. Sary-Dzhaz, 10 Aug 1988, *Pimenov, Kmaishov, Kamsharaeva s.n.* (KRA, FRU); Naryn prov., At-Bashii reg., Ken-Suu r., 15 Aug 1976, *Abgarova s.n.* (KRA); Tian’-Shan’, dolina r. Kuelyu, prav. prit. Sary-Dzhasa, 20–30 Jul 1902, *V. Sapozhnikov s.n.* (LE, TK); Dzhety-Oguzovskii region, Akshiryak, 6–7 km NE of Uzun River, terrace of Cholak River, alt. 3800 m, high mountain steppe, 26 Jul 1935, *I Sanych, G. Sabardina 121* (BM 1191534); Dolina Aksya, Poima r. Terek, Ken-Suu, 1960, *Makarenko s.n.* (FRU, KRA); Semirech. obl. Przhev. u r. Inyl’chek, v 10 v. ot’ lednika, 1 Aug 1912, *V. Sapozhnikov, B. Shishkin s.n*. (LE); C Tian’-Shan’, verkhie Narynskie syrty, ur. Kum-tala, 23 Aug 1926, *R.I. Abolin 1034* (LE); Semirech. obl. Przhev. u r. Sarydzhas’, pri usti r. Myntur’, syrty, 28 Jul 1912, *V. Sapozhnikov, B. Shishkin s.n*. (LE, TK, AA); Tian’-shanskaya obl., Terskei khr., pravyi bereg r. Sary-dzhaz, uste r. Kuisyu, 19 Jul 1967, *Pavlova s.n.* (LE); Issykulskaya obl. Pokrovskie syrty, Aug 1957, *Moldoyarov s.n.* (LE); Semirech. obl. Przhev. u r. Dzhangart’ srednee techenie, travyanistye sklony, 31 Jul 1913, *B. Shishkin s.n*. (TK); Semirech. obl. Przhev. u Kara-Archa, kamenistye sklony, 6 Aug 1912, *V. Sapozhnikov, B. Shishkin s.n*. (TK); C Tian’-Shan’, Khr. Bornokoi, basein r. Karakol, alt. 3700 m, 1949, *M. Tlo[..] s.n.* (AA); Tyan’-shanskaya obl. At-bashinsii raion, bass. r. Ak-saya, stats. Cykanova, Jul 1972, *Nemetskaya s.n.* (FRU); Atbashinskii raion, dolina Ak-sai, pologie sklony, 31 Jul 1939, *Gusarova s.n.* (FRU). **Tajikistan**: Pamir, Kara-kul, dol. Kara-Art, v 5 km ot ust’ya na terrase, alt. 4050 m, 28 Aug 1962, *S.S. Ikonnikov* 14896 (LE, KRA 479099); Pamir, ad lacum Kara-kul, alt. 13500 ft, 5 Jul 1901, *Alexeenko 1392* (LE); Pamir, Shad-Put, peski nizovev dol. r. Up-turukh, alt. 4000 m, 19 Jul 1945, *I. Raikova 159* (LE); Pamir, Rang-Kulskii c/c, 1952, *Sudorov s.n.* (LE); SE Pamir, zap. sklon Ak-tash, Shaimak settl., 29 Aug 1953, *S. Ikonnikov s.n*. (LE); Shot-put, Rang-Kulkii c/c, 20 Jul 1945, *I. Raikova 161* (LE); Pamir, Kara-kul’, po krayu lugov u reki Kara-art, 20 Aug 1955, *Sudorov s.n.* (LE). **Pakistan**: S side of Pamir Pass, near Shuwart, alpine steppe, dominated by Gramineae and flat cushions of *Oxytropis* spp., pastures and flushes, grazed, 36°23–25'N, 75°41–43'E, alt. 4420–4520 m, 16 Aug 1991, *G. & S. Miehe 6173* (GOET); Upper Braldu tributary, above Chikor, alpine steppe, dominated by Gramineae and flat cushion of *Oxytropis* spp., 36°22–24'N, 75°22–24'E, alt. 4220 m, 17 Aug 1991, *G. & S. Miehe 6209* (GOET); Khunjerab-pass, alpine steppe, 36°50'N, 75°25'E, alt. 4460 m, 18 Aug 1990, *G. & S. Miehe 2495* (MSB 154127). **India**: NW India, Jammu and Kashmir State, Ladakh, Zanskar, Markha, Hankar Village to Zalung Karpo La, W slopes of Kyangze, alt. 4700 m, 20 Aug1998, 33°44,5'N, 77°29'E, *L. Klimeš* 86 (KRA 479097). **China: Xinjiang province**, Qira Xian, Nor, Yamei, on slope grassland, alt. ca. 3100 m, 3 Jul 1988, *S. Wu, H. Ohba, Y. Wu, Y. Fei 2532* (MOIS 4374351); Qiemo Xian, Kongqibulaker, on desert grassland, alt. ca. 3200 m, 19 Jul 1988, *S. Wu, H. Ohba, Y. Wu, Y. Fei 2591* (MOIS 5660759); alt. 4050 m, 8 Aug 1959, *no. 1685* (PE 707259); 26 Aug 1965, no. 39181 (PE 707280); Qiemo Xian, Kongqibuaker, on arid soil slope, alt. ca. 4000 m, 26 Jul 1988, *S. Wu, H. Ohba, Y. Wu, Y. Fei 2103* (MOIS 4364181); Qiemo Xian, Kongqibulaker, on sparsely grass-covered hill slope, alt. ca. 3150 m, 19 Jul 1988, *S. Wu, H. Ohba, Y. Wu, Y. Fei 2056* (MOIS 4373368); SW Xinjiang, Karakoram, Aghil Shan northern declivity, ca. 7 km northwest of Aghil Pass, gravelly slope, limestone, 36°14'N, 76°34'E, alt. 4200 m, 30 Aug 1986, *B. Dickoré 487* (GOET); SW Xinjiang, Karakoram, Aghil Shan northern declivity, Aghil Valley, at the Kirghiz summer settlement, ca. 19 km SW of Ylik (Yarkand), north-facing limestone cliff, in fissures, 36°15'N, 76°33'E, alt. 4150 m, 29 Aug 1986, *B. Dickoré 472* (GOET); SW Xinjiang, Karakoram, Aghil Shan northen declivity, ca. 3 km NWW of Aghil Pass, gravelly slope, between granite boulders, dry alpine “turf”, 36°13'N, 76°35'E, alt. 4520 m, 30 Aug 1986, *B. Dickoré 513* (GOET); Tibet borealis, ozero Orich-nor, yuzhn. bereg, alt. 13500 ft, 18 Jul 1884, *N.M. Przwalski 338* (LE), Kam (Tibet), basein Yan-tszy-tszyan’a (r. Goluboi), 1 Aug 1900, *V.O. Ladygin 434* (LE); Kun-lun, Kashgaria, verkhov’e r. Lapet, 20 Jul 1942, V.I. Serpukhov 474 (LE); Kun-lun, Kashgaria, r. Kara-dshilga, levyi pritok r. Gon-arek, alt. 4000–4500 m, 22 Jul 1942, *V.I. Serpukhov 508* (LE); **Gansu Province**, Yumu Mountain, Dahe District, Sunan County, dry hillside, 5 Aug 1967, *Hexi Team 165* (PE); Kansu, alt. 2600 m, 25 Aug 1967, *no. 274* (PE 707218); Kansu, Richthofen (Nan-Shan), Hung-Shui-Pa-Shang-Ho, alt. ca. 3500 m, 28 Aug 1931, *B. Fries-Johansen 2878* (BM 1031149, 1191537); **Qinghai Province**, Haixi Mongol and Tibetan Autonomous Prefecture, stone gap, alt. 3000 m, 28 Jul 1975, *W. Wong, B. Guo 11742* (PE); near the road G109, 18 km SW of the lake, NE of Dashi Bridge, 3617 m, 36°43'34"N, 99°34'51"E, 24 Jul 2010, *B. Paszko s.n*. (KRA); Mengnan County, Qingshui Town, dry slope, alt. 3100 m, 26 Aug 1975, *W. Wang, Be-z. Guo 12192* (PE); Qaidam, kumirnya Dulan’-Khiti, alt. 10100 ft, 12 Aug 1901, *V.O. Ladygin s.n*. (LE); Yeningou North Mountain, Qilian County, meadow, alt. 3400 m, 5 Sep 1975, *W. Wang, B-z. Guo 12473* (PE 707250); alt. 3700 m, 4 Sep 1975, *W. Wang, B-z. Guo 12411* (PE 707242); alt. 3600 m, 10 Aug 1975, *W. Wang, B-z. Guo 12053* (PE 707240); Dari Xian: Jimai Xian, Huleanma, along Huang, flood plain of Hunag He, tussock grass, 33°43'40"N, 99°21'1"E, alt. 4030 m, 11 Aug 1993, *T-n. Ho, B. Bartholomew 1158* (PE 707267, BM 573601); Shinaihaixiang, alt. 3230 m, 36°59'26,9"N, 99°36'03,0"E, 24 Jul 2010, *B. Paszko s.n*. (KRA); Tibetan Autonomous Prefecture of Haibei, hillside, alt. 3300 m, 29 Aug 1975, *W. Wang, B-z. Guo 12262* (PE 707241); Menyuan County, Gingshizui, dry hillside, alt. 3200 m, 24 Aug 1975, *W. Wang, B-z. Guo 12176* (PE); Qinghai, C Tibet, Tangula Shan N, Upper Yangtse Basin, Bi Qu, Wenquan - Yanshiping (Lhasa – Golmud Rd.), 33°31'N, 91°58'E, alt. 4800 m, 18 Aug 1989, *B. Dickoré 4207* (MSB 152888); Qinghai, C Tibet, Tangula Shan N, Upper Yangtse Basin, Gar Qu Vy. (Mt. Geladandong – Yanshiping), 33°36'N, 91°44'E, alt. 4850 m, 2 Sept 1989, *B. Dickoré 4617* (MSB 152889); Heka area of Xinghai County in Qinghai, dry slope, 35.9 N, 99.9 E, alt. 3300 m, Jul 1965, *P-c. Kuo & T-n. Ho 65-6111* (GOET); Gande (Gadê) Xian, near Shanggongma Xiang, Gande (Gadê) Shan, on road from Dari (Darlag) to Gande (Gadê), flat-bottomed valley with moist alpine meadows on bottom, slopes with rocky outcrops and thick turf, on grassy slope, alt. 4150 m, 33°53'45"N, 99°40'50"E, 9 Aug 1993, *T. Ho, B. Bartholomew & M. Gilbert 952* (E 690603, BM 000573603); Madoi Xian: just E of Malayiwan, on road between Gonghe and Madoi, open *Stipapurpurea* steppe on sandy soil, alt. 4050 m, 35°0'N, 98°30'E, 10 Aug 1996, *T. Ho, B. Bartholomew, M. Watson & M. Gilbert 1585* (E 125866, PE 707271, BM 573604); Maquin (Maqên) Xian, Naheqingma, Youyun Xiang, between Dari (Darlag) and Huashuxua, consolidated sand dunes with disturbed flat areas, alt. 4190 m, 33°18'39"N, 99°10'53"E, 17 Aug 1993, *T. Ho, B. Bartholomew & M. Gilbert 1342* (E 690703, BM 573602); Dari (Darlag) Xian: Huleanma, Jianshe Xiang, S side of the Huang He and SW of confluence with the Dari He (Dar Qu), flood plain of the Huang He, tussock grass, alt. 4030 m, 33°43'N, 99°21'E, 11 Aug 1993, *T. Ho, B. Bartholomew & M. Gilbert 1158* (E 690604); Maquin (Maqên) Xian, Dawu Xiang, along the Gequ He, N of Maquin (Maqên), Jiangrang, side of valley with steep slope, mostly dry with large tussock grasses and bare soil, shallower slopes with alpine meadow, areas of deeper soil with shrubs, on slope, alt. 3500 m, 34°42'28"N, 100°14'39"E, 31 Jul 1993, *T. Ho, B. Bartholomew & M. Gilbert 623* (E 690605, PE 707268, BM 573600); Yushu Xian, Xiao Surmang Xiang, between Jerikug and the Xizang border, alt. 3550–3650 m, 32°6'N, 97°16'E, 24 Aug 1996, *T. Ho, B. Bartholomew, M. Watson & M. Gilbert 2315* (E 61899); Near the camp XLIV in E Tibet, 15 Jul 1901, *Sv. Heidin 5127* (BM 001191536); **Xizang Province**, near Ranwu District, Basu County, alt. 4000 m, 18 Aug 1980, *no. 1231* (PE 707340); Tibet, alt. 5100 m, 27 Aug 1963, *no 1989* (PE 707466).

#### ﻿ Stipa arenosa

Taxon classificationPlantaePoalesPoaceae

(Tzvelev) M. Nobis, P.D. Gudkova, Krzempek & Klichowska, comb. and stat. nov.

15800F4F-E7BB-5C25-B515-18301DEE7B5B

urn:lsid:ipni.org:names:77297806-1

≡ Stipapurpureasubsp.arenosa (Tzvelev) D.F. Cui, Flora Xinjiangensis 6: 307. 1996.

##### Basionym.

Stipapurpureavar.arenosa Tzvelev, Rastenia Tsentral’noi Azii po materialam Botanicheskogo Instituta im. V. L. Komarova 4: 60. 1968.

##### Type protologue.

Tibet bor.-occid., praemontium bor. jugi Przeval’skii, ad 5000 m alt., in steppa arenosa, 24.08.1980, V. Roborovski (LE). **Type**: Thibet boreal.-occid., Kuen-Lun, Khr. Przeval’skogo, severnye peredgorya, 24 Aug 1890, *W.J. Roborowski s.n*. (holotype, LE 01010497!).

##### Description.

***Perennial plant***, densely tufted, with a few culms and numerous vegetative shoots; culms (14.7–)16.2–39.1(–50.0) cm tall, 1–2(–3)-noded, nodes distributed close together and only in the lowermost part of the culm. ***Leaves of vegetative shoots***: sheaths shortly and densely pubescent; ***ligules*** truncate to slightly acute, on the external sheaths (0.5–)1.0–1.8(–2.3) mm long, whereas on the internal sheaths (1.0–)1.5–3.0(–4.5); ***blades*** convolute, green, pale green to glaucous, (4.9–)6.1–11.4(–16.0) cm long, 0.4–0.5 mm in diameter, adaxial surface covered by 0.15–0.2 mm long hairs, abaxial surface glabrous, scabrous or setulose. ***Cauline leaves***: lower sheaths sparsely pubescent to almost glabrous, upper sheath glabrous or almost so; ***ligules*** acute, on the lower sheaths (0.7–)1.2–1.9 mm long, on the middle sheaths (1.6–)1.9–3.5(–4.5) and on the upper 2.0–3.45(–4.5); ***blades*** convolute, green or pale green, (2.5–)3.0–6.5(–7.8) cm long, adaxial surface covered with short hairs, abaxial surface glabrous, scabrous to shortly pubescent. ***Panicle*** (7.6–)8.7–14.4(–17.8) cm long, open, at base enclosed by the sheath of the uppermost leaf or rarely exerted, branches flexuous, setulose or slightly scabrous or glabrous, single or paired. ***Glumes*** subequal (the lower slightly longer than the upper), purplish, glumes (19.5–)21.0–23.5(–25.0) mm long, narrowly lanceolate. ***Floret*** (lemma + callus) (11.5–)12.0–14.0(–15.0) mm long. ***Callus*** (2.0–)2.3–3.0(–3.8) mm long, densely pilose on the ventral part, with hairs (0.4–)0.6–0.9(–1.0) mm long, on dorsal part sparsely and shortly pilose with straight hairs (0.5–)0.6–0.8(–0.9) mm long; callus base 0.5–0.8 mm long and 0.15–0.25 mm wide, sharply pointed, scar narrow-elliptic. ***Lemma*** coriaceous, pale-green, purplish or brownish, covered throughout (from the bottom to top) by dense ascending to appressed hairs 0.2–0.4 mm long; top of lemma surpassed by a ring of unequal hairs 0.4–0.9 mm long. ***Awn*** (78–)95–112(–130) mm long, bigeniculate, straw-coloured to brownish; **first segment of the awn** (column) (7–)11–22 mm long, twisted, 0.3 mm wide at base, with (1.5–)1.6–2.0(–2.3) mm long hairs, **middle segment of the awn** 10–12(–13) mm long, twisted, with (1.7–)1.9–2.4 mm long hairs; **terminal segment of the awn** (seta) arcuate or flexuous (62–)68–89(–98) mm long and, hairs longer than those on columns, (2.1–)2.3–2.5(–3.0) mm long, gradually decreasing in length towards apex. ***Palea*** equalling lemma in length.

##### Habitat.

High mountain steppes, semi-deserts, 3500–5000 m. a.s.l.

##### Distribution.

China: southern Xinjiang, Qinghai, Gansu, north-western Xizang; Fig. 3 (Tzvelev 1968).

##### Selected specimen examined.

**China: Qinghai Province**: Kuen-Lun, Dolina r. Sharagol’-dzhin, yr. Paidza-Tologoi, 11000 ft alt., pesch. step’, 11 Jul 1894, *W.J. Roborowski 318* (LE 01010495 [label 2: Stipapurpurea Griseb f. robusta, det. R. Roshevitz; Label 3: Stipa kozlovii m. sp. nov. inedit. = S.purpurea var. arenosa m. var. nova, Typus varietis!, XI. 1966, N.N. Tzvelev], LE 01010496, KRA 476855, K, - paratypes); **Xinjiang Province**, Ruoqiang, N of Aqqikkol, on grasslands, ca. 4200 m alt., 21 Aug 1988, *S. Wu, H. Ohba, Y. Wu, Y. Fei 2747* (MOIS 3744710); Xinjiang, Ruoqiang, Yueya River to Aqqikkol, in Stipa grassland on gravel-rich flat places, 21 Aug 1988, *S. Wu, H. Ohba, Y. Wu, Y. Fei 2275* (MOIS 5660755); **Gansu Province**, Subei County, alt. 3500 m, 5 Aug 1956, *B-z. Guo s.n*. (PE 707226); **Xizang Province**, 16 km northeast of Shuanghu County, alt. 5000 m, 27 Jul 1976, *Gansu Agricultural University 111* (PE 2029866).

##### Note.

Stipapurpureavar.arenosa was described, based on two collections of W.J. Roborowski from Central Asia (Tzvelev 1968). During our research in 2009–2021, in addition to the holotype, we found four sheets (paratypes) with specimens of *Stipa* collected by Roborowski in 1894. The two of the paratypes preserved in LE, were labeled as the type and isotype of Stipapurpureavar.arenosa by N. Tzvelev in 1966.

### ﻿A new natural hybrid between *S.klimesii* and *S.tremula*

In western Himalayas (Ladakh, NW India), within high mountain steppes and semi-deserts, *S.kilmesii* and *S.tremula* or *S.purpurea* sometimes co-occur and hybridisation events between the species may occur. During taxonomic revision of the Himalayan feathergrass species, we found a putative product of such hybridisation, that was collected by L. Klimeš in Spangchen Do (Ladakh, NW India) in 2001. The putative hybrid taxon is similar to *S.purpurea* in having long and flexuous branches in the lower part of the panicle; however, in comparison to *S.purpurea*, *S.×ladakhensis* has narrower panicles and shorter awns with hairs on the setae equal or shorter than the hairs on the columns (Table 3). The new taxon also differs from the second parental species, *S.klimesii*, by having longer and flexuous branches, longer glumes and somewhat longer awns (Table 3). Following Nobis et al. (2020), over 30% of species within the genus are of hybrid origin. In feathergrasses, the hybrids are perennial and reproduce vegetatively and, less frequently, sexually (Nobis 2013; Nobis et al. 2017, 2020). Most of them produce some fertile pollen grains and, therefore, may be able to backcross with their parental species, resulting in introgression (Nobis et al. 2017, 2019c; Baiakhmetov et al. 2020, 2021). Thus, for better understanding of the microevolution processes, it is important to detect hybrids and hybridisation events in *Stipa*.

#### ﻿ Stipa × ladakhensis

Taxon classificationPlantaePoalesPoaceae

M. Nobis, Klichowska, A. Nowak & P.D. Gudkova, nothosp. nov.(S. klimesii × S. purpurea s.l.)

37EB8891-8E76-53A2-8B7A-1A9517427F3A

Fig. 5

##### Type.

NW India, Jammu and Kashmir State, Ladakh Region: Zanskar: Zara, Spangchen Do, alt. 4520 m, 1 Sep 2001, 33°22.7'N, 77°45.1'E, code 01-34-13, *L. Klimeš 1474* (holotype KRA 603490!, isotypes PR!, KRA 603487!, 603486!)

**Figure 5.**
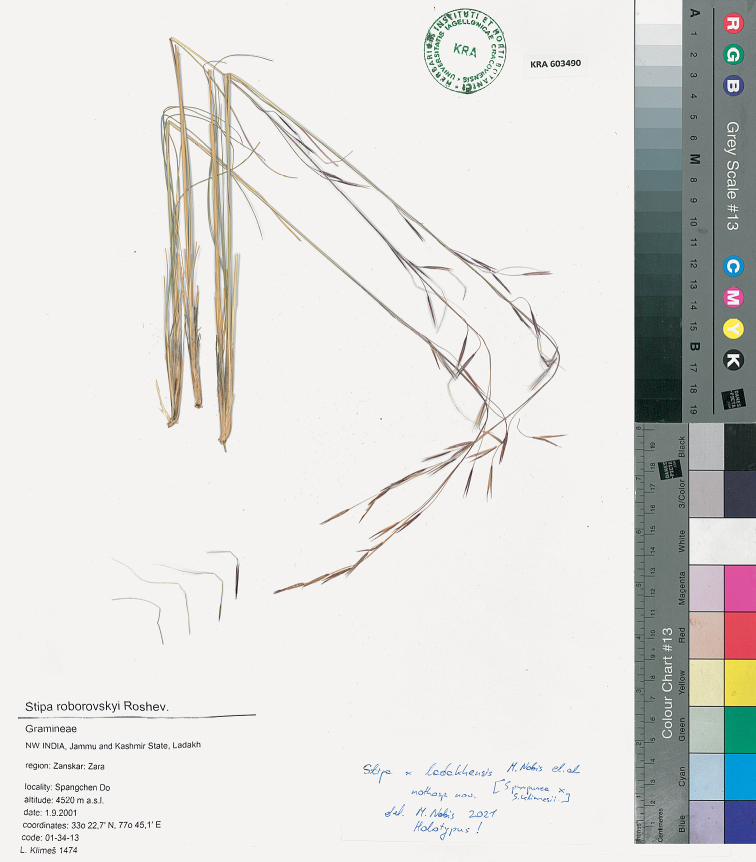
The holotype of *Stipa×ladakhensis* M. Nobis, Klichowska, A. Nowak & P.D. Gudkova.

##### Description.

***Plant perennial***, densely tufted, with a few culms and numerous vegetative shoots; culms 35–55 cm tall, 1–2-noded, nodes distributed close together and only in the lowermost part of the culm. ***Leaves of vegetative shoots***: sheaths shortly and densely pubescent; ***ligules*** acute, on the external sheaths (1.0–)1.2–2.0(–2.8) mm long, whereas, on the internal sheaths (1.5–)2.0–4.0(–5.5) mm long; ***blades*** convolute, green, pale green to glaucous 10–25 cm long, 0.3–0.5 mm in diameter, adaxial surface covered by 0.15–0.2 mm long hairs, abaxial surface scabrous. ***Cauline leaves***: lower sheaths shortly pubescent, upper scabrous or glabrous; ***ligules*** acute, (1.0–)2.0–4.0(–5.0) mm long; ***blades*** of convolute, green or pale green, adaxial surface shortly pubescent, abaxial surface scabrous or glabrous. ***Panicle*** 17–25 cm long, rather contracted, with 15–23 spikelets, at base enclosed by the sheath of the uppermost leaf or exerted, lower branches 2–6 cm long, straight or slightly flexuous, setulose or glabrous, single or paired. ***Glumes*** subequal, brownish to purplish, glumes 16–19(–20) mm long, narrowly lanceolate, tapering into long hyaline apex. ***Floret*** (lemma + callus) 8.5–10.2 mm long and 0.7–0.9 mm wide. ***Callus*** 1.5–1.9 mm long, densely and long-pilose, the base of callus narrow, peripheral ring 0.15–0.20 mm in diameter, acute, scar narrow elliptic. ***Lemma*** coriaceous, straw-coloured, brownish or purplish; covered throughout (from the bottom to top) by dense ascending to appressed hairs 0.2–0.4 mm long. ***Awn*** 46–58 mm long, bigeniculate, **lower segment of the awn** 8–11 mm long, twisted, with 1.7–2.3 mm long hairs, **middle segment of the awn** 4–9 mm long, twisted, with 1.6–2.2 mm long hairs; **terminal segment of the awn** (seta) flexuous 30–42 mm long with hairs shorter to equal to those on the column, 1.6–2.1 mm long, gradually decreasing in length towards the apex. *Palea* equalling lemma in length.

##### Habitat.

High mountain semi-deserts, on the elevation from 4000 to 5000 m.

##### Distribution.

W Himalayas (NW India).

## Supplementary Material

XML Treatment for Stipa purpurea

XML Treatment for Stipa tremula

XML Treatment for Stipa arenosa

XML Treatment for Stipa × ladakhensis
